# Low-grade fibromyxoid sarcoma, a rare tumor at an unusual site: Case report and review of literature

**DOI:** 10.15190/d.2025.8

**Published:** 2025-06-30

**Authors:** Rasheeda Mohamedali, Nilay Nishith, Rahul Raj, Aishwarya Sharma, Puneet Kaur Somal, Ravikiran N Pawar, Sankalp Sancheti, Deepander Singh Rathore

**Affiliations:** ^1^Department of Onco-Pathology, Homi Bhabha Cancer Hospital and Research Centre, Tata Memorial Centre, Homi Bhabha National Institute (HBNI), New Chandigarh, Punjab, India; ^2^Department of Radiodiagnosis, Homi Bhabha Cancer Hospital and Research Centre, Tata Memorial Centre, Homi Bhabha National Institute (HBNI), New Chandigarh, Punjab, India

**Keywords:** Low-grade fibromyxoid sarcoma, MUC4, pleura, rare.

## Abstract

Low-grade fibromyxoid sarcoma (LGFMS) is a rare fibroblastic neoplasm with an indolent clinical course. It is a distinctive subclass of soft tissue sarcoma with metastasizing potential and sometimes a long interval between tumor presentation and metastasis. This case report describes a 60-year-old female with an unresectable pleural LGFMS initially misdiagnosed as malignant mesothelioma. Pleural LGFMS remains exceedingly rare, with only four prior cases reported. Given its histologically benign appearance, LGFMS poses diagnostic challenges and risks of local recurrence or metastasis. This case underscores the importance of accurate diagnosis using MUC4 and it explores endocrine therapy as a promising palliative option for unresectable LGFMS, contributing valuable insights into management strategies for this rare entity.

## INTRODUCTION

Low-grade fibromyxoid sarcoma (LGFMS), also known as the Evans tumor, is a rare fibroblastic neoplasm^1^. These sarcomas tend to affect young individuals and present as a slow-growing mass. The classic sites involved include the upper and lower extremities and the inguinal region, rarely involving the thorax and viscera^2^. Due to its histologically bland appearance, with spindle cells arranged in alternating fibrous and myxoid stroma, LGFMS has a deceptive benign look. This makes it prone to underreporting, potentially leading to local recurrence and distant metastasis. MUC4 is often expressed in LGFMS^3^. Furthermore, LGFMS mostly harbors FUS::CREB3L1/2 gene fusions, and EWSR1::CREB3L1 gene fusion has also been reported^4^. Its occurrence in the mediastinum is rare, and only a handful of cases have been reported in the literature^1,4-12^.

The origin of LGFMS from pleura is all the more unusual^13-15^. Diagnosing LGFMS in this location is challenging and requires an amalgamation of a thorough history, a detailed clinical examination, and various imaging techniques. Even after such an elaborate investigation, the diagnosis of LGFMS can often be misleading. Systemic chemotherapy or radiation is ineffective for LGFMS owing to its low nuclear grade. Thus, a complete surgical resection is performed as part of the standard treatment regimen^12^. We report the case of a 60-year-old female with a pleural-based mass that was clinically diagnosed as malignant mesothelioma. We have tried to probe into treatment options for unresectable LGFMS, like ours, to identify the most effective approach in the modern era.

## CASE REPORT

A 60-year-old female presented with a history of fall and trauma to the head. She had a history of fever with chills, shortness of breath, and an episode of blood in vomitus for the last two days and was admitted to a private hospital for the same. She also had a history of on-and-off hemoptysis for 40 years, for which she was never evaluated. Her father and first-degree relative had carcinoma of the lung and esophagus, respectively.

**Figure 1:**
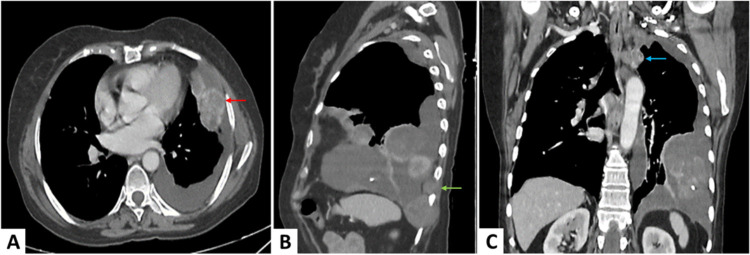
CECT chest A. Axial image showing a heterogeneously, avidly enhancing lobulated pleural-based mass lesion in the left lingular lobe (red arrow), with associated pleural effusion, B. Sagittal image showing mass effect over the left diaphragm and chest wall invasion (green arrow), C. Coronal image of the chest showing lobulated pleural-based mass lesions involving both mediastinal and costal pleura (blue arrow).

The patient underwent a contrast-enhanced computerized tomography (CECT) of the chest, which illustrated multiple irregular, heterogeneously enhancing nodular pleural deposits and masses noted in the left costal, mediastinal, and diaphragmatic pleura, with the largest measuring approximately 6.8 x 5.4 cm along the diaphragmatic pleura. There was a loss of fat planes between the lesions and the intercostal muscles at the level of the 7th, 8th, and 9th ribs on the left side, although no erosion is seen in the overlying ribs. Mild left pleural effusion is also present. Additionally, a few centrilobular emphysematous changes are seen in both lungs, along with a few fibroatelectatic changes in the left lower lobe. Multiple enlarged, indeterminate mediastinal lymph nodes are observed in the paratracheal, aortopulmonary window, carinal, and subcarinal locations, with the largest measuring approximately 17 x 13 mm in the upper left paratracheal region. The trachea and main stem bronchi appear normal, as do the heart and mediastinal great vessels. Based on these radiological findings, the possibility of malignant mesothelioma was suggested (Figure 1A-C). A CT brain was also performed, which showed multiple small wedge-shaped hypodense areas in the left frontal lobe in parasagittal regions, suggestive of chronic infarcts.

**Figure 2:**
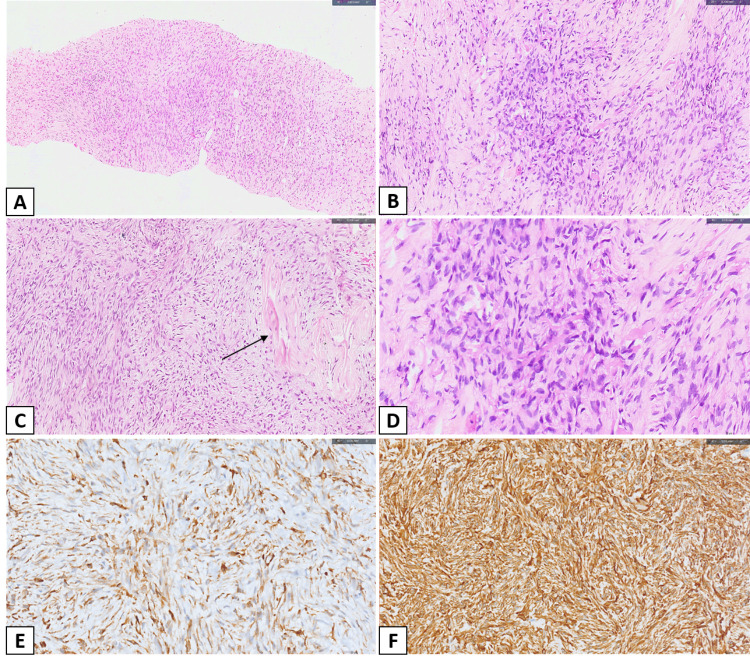
Histopathological findings A-B. Low power magnification showing a mesenchymal neoplasm with alternating myxoid and collagenous areas and disposed as short fascicles (H&E: A. x100, B. x200), C. An area demonstrating vague collagenous rosettes (black arrow) surrounded by neoplastic cells and thin-walled vascular channels (H&E, x200), D. High-power magnification showing spindle to stellate cells with bland nuclei and scant eosinophilic cytoplasm (H&E, x400), E-F. Immunohistochemical findings: E. Cytoplasmic staining of neoplastic cells with MUC4 (IHC, x400), F. Cytoplasmic staining of neoplastic cells with vimentin (IHC, x400).

A CT-guided biopsy was performed from the diaphragmatic pleural deposit. The section showed linear cores revealing a mesenchymal neoplasm with alternating myxoid and collagenized areas (Figure 2A) composed of spindle to stellate cells arranged as short fascicles and focal whorling pattern (Figure 2B). The neoplastic cells have bland nuclei with scant eosinophilic cytoplasm. These cells are embedded in fibrous to myxoid stroma with focal collagenization (Figure 2C-D). A few scattered thin-walled vascular channels are also noted. Mitotic figures are inconspicuous. Necrosis is absent. By immunohistochemistry, the neoplastic cells are positive for MUC4 (Figure 2E) vimentin (Figure 2F), focally positive for CD99, and BCL2, while they are negative for Calretinin, WT1, Desmin, SMA, CD34, S100, AE1/AE3, CK5/6, BerEP4, h-Caldesmon, EMA, beta-catenin, TLE1. MIB-1 labeling index is 1-2% in the highest proliferating area. A final diagnosis of low-grade fibromyxoid sarcoma was rendered. To further substantiate the diagnosis, the patient was advised to undergo analysis for FUS gene rearrangement but was unable to do so due to financial constraints.

Since the mass was unresectable, the patient opted for palliative systemic therapy. The patient was started on hormonal drug therapy (Tamoxifen and Letrozole) in view of immunohistochemistry ER (estrogen receptor) and PR (progesterone receptor) positivity.

**Table 1:** Clinicopathological features of cases reported as low-grade fibromyxoid sarcoma with primary pleural involvement

Reference with the year of publication	Age/Sex	Tumor size (in cm)	Pleural effusion	Histological findings	Management	Outcome
Kim et al, 2005^15^	37/M	Not reported	Not mentioned	Tumor composed of fibrous parts, which showed bland spindle-shaped cells. In myxoid zones, cells were spindle to stellate type	Not mentioned	Not reported
Liang et al, 2014^14^	42/F	10 x 11	Present	Spindle tumor cells are arranged in a whirlpool-like structure	Incomplete surgical resection and radiotherapy	Not reported
Perez et al, 2020^16^	32/M	11.0 (single mass with multiple chest wall deposits)	Present	Uniform bland-looking spindle cells intermingled with hyalinized rosettes	Surgical resection	No local recurrence or metastasis in 29 months of follow-up
He et al, 2023^7^	4/M	2.2 x 1.9	Not mentioned	Fibrous stroma and myxoid areas with spindle cells arranged in a whorled and short fascicular pattern	Surgical resection	No recurrence in 11 months of follow-up
Index case	60/F	6.8 x 5.4	Present	Spindle to stellate cells arranged as short fascicles and focal whorling pattern, embedded in alternating myxoid and collagenous stroma.	No surgical intervention; Started on endocrine therapy	Stable disease for the last 10 months

She has been on a follow-up every three months for the past 10 months, and the disease is stable.

## DISCUSSION

LGFMS was originally described as a painless lump in the soft tissue around the scapula, axillary, and chest areas by Evans in 1987^1^. Sarcomas account for 1% of adult cancers, and LGFMS is estimated to represent fewer than 5% of soft-tissue sarcomas^2^. The incidence of LGFMS was estimated to be 0.18 per million^13^. This rare sarcoma mainly involves the deep soft tissue of the proximal extremities and trunk but can occur at rare sites like the mediastinum and viscera, too. The occurrence of LGFMS in the pleura is exceedingly rare and has been reported only in four cases (Table 1)^7,14-16^.

Regardless of its histologically bland appearance, LGFMS occasionally recurs locally or metastasizes to the lung^1^. Histologically, LGFMS cells are spindle-shaped with alternating fibrous and myxoid regions, sometimes with a nodular pattern and often with an abrupt transition from one pattern to another. The myxoid areas typically show prominence of small vessels with branching, described as curvilinear or arcades. The fibrous areas vary from moderately to extremely hypocellular. Cellularity overall varies from low to moderate but never high, as in a ‘blue’ tumor. The tumor cells vary from spindle to stellate. Nuclei are medium-sized and extremely regular. Mitosis and necrosis are very uncommon. Cytoplasm is scanty. As LGFMS has a diverse histological appearance, it is difficult to distinguish it from other sarcomas and benign tumors^1,17,18^.

LGFMS is particularly difficult to distinguish from sclerosing epithelioid fibrosarcoma (SEF). The latter occurs in the middle to elderly age group and carries a poor prognosis. Although both tumors are interrelated, SEF tends to behave more aggressively^18^.The other differential diagnoses to be considered while diagnosing LGFMS include myxoid tumors such as myxomas, angiomyxomas, myxofibrosarcoma, and myxoid liposarcoma. Myxomas and angiomyxomas lack fibrous tissue and have lower vascularity. Myxofibrosarcoma typically affects the elderly and exhibits higher levels of nuclear pleomorphism. Liposarcomas usually have lipoblasts with prominent vacuolated cytoplasm. The closest fibrous tumor showing bland spindle cells with a locally infiltrative pattern is fibromatosis. Although they do not show any curvilinear blood vessels or an alternating myxoid stroma, most cases are diagnosed by beta-catenin^1,17,18^.

The spindle cell lesions that may exhibit both myxoid and fibrous stroma are difficult to negate, especially in core biopsies. These include neurofibroma, malignant peripheral nerve sheath tumor, perineuroma, fibrous histiocytoma, nodular fasciitis, and solitary fibrous tumor. With a careful histological evaluation and judicious use of immunostains, a diagnosis of LGFMS cannot be missed. Immunohistochemistry is consistently positive for vimentin only, and negative or only positive in occasional cells with a variety of antibodies, such as SMA, S100, EMA, and desmin^1,17,18^. MUC4 is currently considered to be the most specific and sensitive indicator of LGFMS. It is diffusely and strongly positive in LGFMS^3^. This tumor is genetically described by chromosomal translocations involving the FUS gene, most commonly as FUS-CREB3L2 (75%–95%) and FUS-CREB3L1 (5%–10%)^4^. However, it is pertinent to note that as per the WHO Blue Book 5^th^ edition, the FUS gene rearrangement is a desirable diagnostic criterion rather than an essential one and should only be considered in cases of MUC4 negativity^18^.

LGFMS remains challenging to treat, particularly in advanced or metastatic stages. Current standard care for localized disease involves complete surgical resection with clear margins, achieving 10-year overall survival rates near 100%^2,19^. Adjuvant radiotherapy is occasionally used for extremity tumors to improve local control, though LGFMS is not considered highly radiosensitive^2^. For recurrent/metastatic cases, conventional chemotherapy shows minimal efficacy, with a 0% RECIST 1.1 response rate and median progression-free survival of 1.84 months^2^. Future directions focus on molecular-targeted therapies and clinical trials. The *FUS-CREB3L2/L1* fusion genes present potential therapeutic targets, with early-phase trials (e.g., PAZANTIS, TRASTS) investigating pazopanib and trabectedin-radiotherapy combinations.^2^ Evans et al. reported 33 cases of LGFMS, with local recurrence in 21 and metastasis in 15. Recurrence occurred up to 15 years (median 3.5 years), while metastasis appeared as late as 45 years (median 5 years). Fourteen patients died after up to 42 years (median 15 years), and six were alive with the tumor at follow-up extending to 70 years (median 17 years). These findings highlight the tumor’s potential for late recurrence, metastasis, and prolonged clinical course, emphasizing the need for long-term follow-up^20^.

In the index case, the radiological findings clearly show that the tumor had invaded the intercostal muscles, indicating that the disease had been long-standing. This delay in seeking timely medical attention contributed to its progression, and therefore, it was deemed unresectable. Subsequently, a thorough literature search was conducted to identify the best possible treatment option for the patient. The review indicated that a handful of authors have advocated a role for hormonal therapy in metastatic or recurrent LGFMS^2,13^. Accordingly, based on the immunopositivity of ER and PR, endocrine therapy was offered as palliative management. The patient has been on Tamoxifen and Letrozole for the past 10 months with follow-up every three months. The disease remains stable to date.

## CONCLUSION

LGFMS is a highly heterogeneous subclass of soft tissue sarcoma with unique molecular characteristics. We report a rare case of primary pleural LGFMS along with a literature review of a handful of published cases. The experience of most pathologists with this tumor is limited because of its rarity and the extremely long interval between tumor presentation and metastasis. To reach a definitive diagnosis, FUS gene translocation is useful. However, MUC4 is the most specific marker and is useful even in FUS wild-type. Surgical resection remains the treatment of choice for LGFMS. Nonetheless, endocrine therapy may be considered as a palliative option in cases of unresectable, recurrent, or metastatic disease. Thus, this case report underscores the critical need for accurate diagnosis and innovative treatment approaches in managing rare, unresectable pleural LGFMS, enhancing clinical decision-making.

## Bullet Points

· *Rare pleural LGFMS case expands limited literature on unusual sites and underscores MUC4’s role in accurate diagnosis against benign-appearing spindle tumors*

· *ER/PR‑targeted endocrine therapy offers a novel palliative option for unresectable LGFMS*

· *LGFMS is an indolent yet persistent neoplasm that necessitates prolonged surveillance for late recurrence or metastasis*
